# Different effects of deep inspirations on central and peripheral airways in healthy and allergen-challenged mice

**DOI:** 10.1186/1465-9921-9-23

**Published:** 2008-02-28

**Authors:** Sofia Jonasson, Linda Swedin, Maria Lundqvist, Göran Hedenstierna, Sven-Erik Dahlén, Josephine Hjoberg

**Affiliations:** 1 Department of Medical Sciences, Clinical Physiology, Uppsala University, Uppsala, Sweden; 2 The National Institute of Environmental Medicine, Division of Physiology, Karolinska Institutet, Stockholm, Sweden

## Abstract

**Background:**

Deep inspirations (DI) have bronchodilatory and bronchoprotective effects in healthy human subjects, but these effects appear to be absent in asthmatic lungs. We have characterized the effects of DI on lung mechanics during mechanical ventilation in healthy mice and in a murine model of acute and chronic airway inflammation.

**Methods:**

Balb/c mice were sensitized to ovalbumin (OVA) and exposed to nebulized OVA for 1 week or 12 weeks. Control mice were challenged with PBS. Mice were randomly selected to receive DI, which were given twice during the minute before assessment of lung mechanics.

**Results:**

DI protected against bronchoconstriction of central airways in healthy mice and in mice with acute airway inflammation, but not when OVA-induced chronic inflammation was present. DI reduced lung resistance induced by methacholine from 3.8 ± 0.3 to 2.8 ± 0.1 cmH_2_O·s·mL^-1 ^in healthy mice and 5.1 ± 0.3 to 3.5 ± 0.3 cmH_2_O·s·mL^-1 ^in acute airway inflammation (both *P *< 0.001). In healthy mice, DI reduced the maximum decrease in lung compliance from 15.9 ± 1.5% to 5.6 ± 0.6% (*P *< 0.0001). This protective effect was even more pronounced in mice with chronic inflammation where DI attenuated maximum decrease in compliance from 44.1 ± 6.6% to 14.3 ± 1.3% (*P *< 0.001). DI largely prevented increased peripheral tissue damping (G) and tissue elastance (H) in both healthy (G and H both *P *< 0.0001) and chronic allergen-treated animals (G and H both *P *< 0.0001).

**Conclusion:**

We have tested a mouse model of potential value for defining mechanisms and sites of action of DI in healthy and asthmatic human subjects. Our current results point to potent protective effects of DI on peripheral parts of chronically inflamed murine lungs and that the presence of DI may blunt airway hyperreactivity.

## Background

Mice are increasingly being used to develop *in vivo *models for studying airway physiology and airway inflammation. Exposure to aerosolized antigen in animals mimics the chronic inflammatory characteristics of human asthma and prolonged exposure to allergen has been suggested to be of importance for the development of airway hyperreactivity and remodeling in asthma [1,2].

Deep inspirations (DI) have been shown in human subjects to cause a decrease in airway resistance, to have bronchoprotective effects in healthy subjects, and to reverse bronchoconstriction [3-8]. The effectiveness of a deep inspiration is related to the number of DI before administration of a bronchoconstricting stimulus [4]. There is convincing evidence that both bronchodilatory and bronchoprotective actions of DI are deficient or absent in the asthmatic lung and it has been proposed that a lack of bronchoprotective or bronchodilatory effects of DI may play a major role as an underlying abnormality leading to airway hyperreactivity in asthma [5,7,9-13].

In this study, we aimed at characterizing the effects of DI on lung mechanics during mechanical ventilation in healthy mice and in mice exposed to allergen to simulate asthma and we describe both a murine OVA model for acute inflammation and a model for chronic inflammation that may resemble chronic airway inflammation in humans. Our goals were to investigate if these mouse models could be used to identify the site of action of DI and whether it is a good model of response to DI in normal and asthmatic subjects.

## Methods

### Animals

Female Balb/c mice (Charles River, Sulzfeld, Germany, and Taconic (M&B), Denmark) were used in this study. They were housed in plastic cages with absorbent bedding material and were maintained on a 12 h daylight cycle. Food and water were provided ad libitum. Their care and the experimental protocols were approved by the Regional Ethics Committee on Animal Experiments in Sweden (Stockholm N348/05 and Uppsala C86/5). Healthy mice were 12 weeks of age and weighed 20.5 ± 0.2 g and animals included in the acute airway inflammation study were 9 weeks of age and weighed 18.9 ± 0.2 g when airway physiology was assessed. Animals included in the chronic airway inflammation study were 8 weeks old when the inflammatory protocol started and 22 weeks old and weighed 22.0 ± 0.2 g when airway physiology was assessed.

### Preparation of animals

The mice were anesthetized with an intraperitoneal (i.p.) injection of pentobarbital sodium (90 mg·kg^-1^, from local suppliers). They were tracheostomized with an 18-gauge cannula and mechanically ventilated in a quasi-sinusoidal fashion with a small animal ventilator (FlexiVent^®^, Scireq, Montreal, PQ, Canada) at a frequency of 2.5 Hz and a tidal volume (V_T_) of 12 mL·kg^-1 ^body weight. Once ventilation was established bilateral holes were cut in the chest wall so that pleural pressure would equal body surface pressure and so that the rib cage would not interfere with lung movement. This enabled strict lung mechanics measurements. Positive end-expiratory pressure (PEEP) of 3 cmH_2_O was applied by submerging the expiratory line in water. Four sigh maneuvers at three times the tidal volume were performed when beginning the experiment to establish stable baseline lung mechanics and ensure a similar volume history before the experiments. The lateral tail vein was cannulated for intravenous (i.v.) injections. The mice were then allowed a five min resting period before the experiment began.

### Analysis of lung mechanics

Dynamic lung mechanics were measured by applying a sinusoidal standardized breath and analyzed using the single compartment model and multiple linear regression, giving us lung resistance (R_L_) and compliance (C_L_) [14]. More thorough evaluations of lung mechanics were made using Forced Oscillation Technique (FOT). During the forced oscillatory maneuver the ventilator piston delivers 19 superimposed sinusoidal frequencies, ranging from 0.25 to 19.625 Hz, during 4 s (prime 4), at the mouse's airway opening. Harmonic distortion in the system is avoided by using mutually prime frequencies [15]. Knowing the dynamic calibration signal characteristics, the Fourier transformations of the recordings of pressure and volume displacement within the ventilator cylinder can be used (P_cyl _and V_cyl_) to calculate the respiratory system input impedance (Zrs) [16]. Fitting the Zrs to an advanced model of respiratory mechanics, the constant phase model [15], allows partitioning of lung mechanics into central and peripheral components. The primary parameters obtained are the Newtonian resistance (R_N_), a close approximation of resistance in the central airways; tissue damping (G), closely related to tissue resistance and reflecting energy dissipation in the lung tissues; and tissue elastance (H), characterizing tissue stiffness and reflecting energy storage in the tissues [14,17-19].

### Experimental Protocols

**Figure 1:**
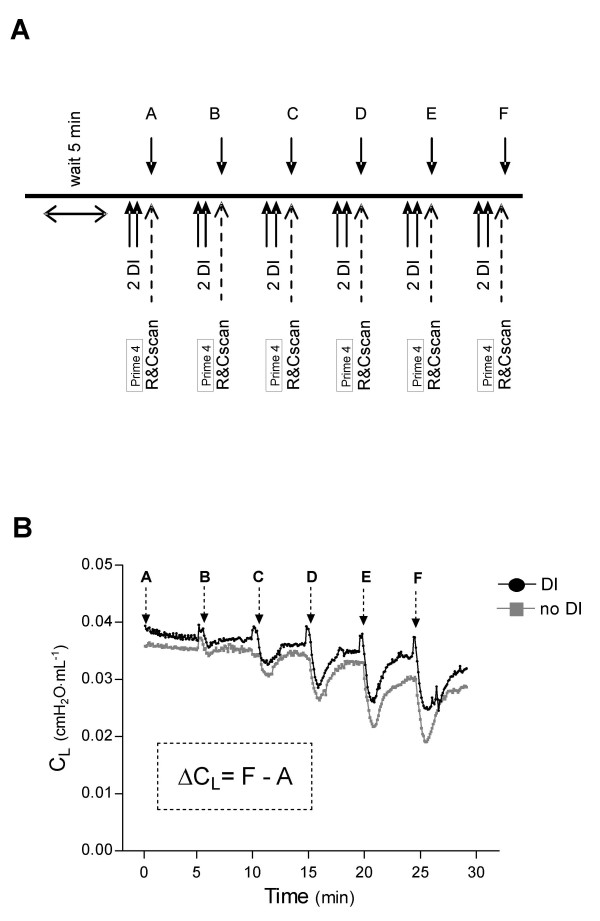
Schematic presentation of study design and graph describing tracings and measurements of lung compliance. (**A**) Experimental protocol. R&Cscan is a program for measuring lung resistance and compliance with the single compartment model. A perturbation of forced oscillation was performed for 4 s (Prime 4, Zrs measurements) and was used in the acute 17-day (OVA'17 and PBS'17 animals) and chronic 98-day protocol (OVA'98 and PBS'98 animals). During A → F, methacholine (MCh) or phosphate buffered saline (PBS) was administrated or nothing was given. MCh or PBS was administrated 20 s after last DI. (**B**) Tracings of lung compliance (C_L_) obtained by R&Cscan indicating measurement points for C_L _(A → F) and ΔC_L _with and without deep inspirations (DI).

Common for all mice studied, lung mechanics measurements were assessed every fifth min during a 30 min protocol (Figure 1A). Mice were randomly selected to receive DI, that was given twice during the minute before assessment of lung mechanics, DI is defined as incremental increase and decrease of three times V_T _during a period of 16 s. Mice not receiving DI, were given normal ventilation for 16 s.

#### Healthy mice

Healthy mice were allocated into the following groups:

1) the TIME group: To investigate the effect of time, lung mechanics were assessed at five min intervals in mice randomly selected to receive DI (TIME+DI, n = 6) or no DI (TIME, n = 6).

2) the PBS group: This group received i.v. injections of 2000 μL·kg^-1 ^phosphate buffered saline (PBS, pH 7.4, Sigma-Aldrich, St. Louis, MO, USA) containing 10 U·ml^-1 ^of heparin. Mice either received DI before each injection and measurement of lung mechanics (PBS+DI, n = 6), or received no DI (PBS, n = 6). PBS was given six times, at five min intervals, lung mechanics were measured immediately before and after the injections at the same time points used for the TIME group.

3) the MCH group: To assess airway responsiveness this group was given incremental doses of MCh (MCh, acetyl-β-methylcholine chloride, Sigma-Aldrich) i.v. (0 = PBS, 0.03, 0.1, 0.3, 1, and 3 mg·kg^-1^) at five min intervals. MCh was diluted in PBS with 10 U·ml^-1 ^of heparin, and a volume of 2000 μL·kg^-1 ^was given at each injection. Lung mechanics were measured immediately before and after the injections at the same time points used for the TIME and PBS groups. Control mice received no DI before the MCh doses (MCH, n = 8), while another group of mice received DI before the injection of MCh (MCH+DI, n = 6).

R_L _and C_L _were measured immediately after each DI or normal ventilation. To further evaluate the ability of DI to reverse a fall in C_L_, we calculated the total fall from baseline to the last measurement of C_L_, denoted ΔC_L _(Figure 1B).

#### Acute allergen-challenged, OVA- or PBS-treated mice

Acute airway inflammation was induced by intraperitoneal injections of 10 μg ovalbumin (OVA, Sigma-Aldrich) emulsified in Al(OH)_3 _(Sigma-Aldrich) on day 0 and day 7. Mice were then challenged with 1% OVA diluted in phosphate-buffered saline (PBS, Sigma-Aldrich). Animals were exposed to aerosolized OVA for 30 min, on day 14, 15 and 16. Aerosol exposure was performed in a chamber coupled to a nebulizer (DeVilbiss UltraNeb^®^, Sunrise Medical Ltd, U.K.). The chamber was divided into pie-shaped compartments with individual boxes for each animal, providing equal and simultaneous exposure to allergen. The experiment ended with assessment of lung mechanics on day 17, 24 h after last allergen exposure. Control mice were sensitized with OVA i.p. and challenged with aerosolized PBS using the same protocol as for OVA described above.

The effects of DI on lung mechanics were investigated after the 17-day protocol in OVA and PBS challenged mice in a fashion similar to that described above for healthy unchallenged mice in the MCH group. Besides, OVA and PBS challenged mice received immediately after each DI or normal ventilation for 16 s, a shorter 4 s perturbation of forced oscillation (Prime 4), followed by the injection. Mice were given one of four treatments:

1) PBS-challenged mice that were given DI (PBS'17+DI, n = 8) before injection of incremental doses of MCh i.v. (from 0 to 3 mg·kg^-1^).

2) Another group of PBS-challenged mice that did not receive any DI (PBS'17, n = 7).

3) OVA-challenged mice that were given DI (OVA'17+DI, n = 8) before injection of incremental doses of MCh i.v. (from 0 to 3 mg·kg^-1^).

4) Another group of OVA-challenged mice that did not receive any DI (OVA'17, n = 10).

#### Chronic allergen-challenged, OVA- or PBS-treated mice

Chronic airway inflammation was induced using the same protocol as for acute OVA described above. However, animals were exposed to aerosolized OVA for 30 min, three days a week between day 14 and 93. Five days after last allergen exposure, the experiment ended with assessment of lung mechanics on day 98. Control mice were sensitized using the same protocol as for acute OVA described above and challenged with aerosolized PBS.

The effect of DI on lung mechanics were investigated after the 98-day protocol in OVA and PBS-challenged mice in a fashion similar to that described above for healthy unchallenged mice in the MCH group. Besides, OVA and PBS challenged mice also received a shorter 4 s perturbation of forced oscillation (Prime 4), followed by the injection. Mice were given one of four treatments:

1) PBS-challenged mice that were given DI (PBS'98+DI, n = 5) before injection of incremental doses of MCh i.v. (from 0 to 3 mg·kg^-1^).

2) Another group of PBS-challenged mice that did not receive any DI (PBS'98, n = 6).

3) OVA-challenged mice that were given DI (OVA'98+DI, n = 5) before injection of incremental doses of MCh i.v. (from 0 to 3 mg·kg^-1^).

4) Another group of OVA-challenged mice that did not receive any DI (OVA'98, n = 6).

### Bronchoalveolar lavage

After completion of the lung mechanics experiment, mice subjected to the 17-day and the 98-day protocol respectively were exsanguinated and subjected to bronchoalveolar lavage (BAL). The lungs were lavaged three times via the tracheal tube with a total volume of 1 mL PBS containing 0.6 mM EDTA (EDTA, Ethylenediaminetetraacetic acid, Sigma-Aldrich). The BAL fluid was then immediately centrifuged (10 min, 4°C, 1200 rpm). After removing the supernatant, the cell pellet was resuspended in 100 μL of red cell lysis buffer containing 0.15 M NH_4_Cl, 1.0 mM KHCO_3_, and 0.1 mM EDTA for 2 min at room temperature. The suspension was then diluted with 1 mL PBS and recentrifuged (10 min, 4°C, 1200 rpm). Leukocytes were counted manually in a hemacytometer so that 50,000 cells could be loaded and centrifuged using a cytospin centrifuge. Cytocentrifuged preparations were stained with May-Grünwald-Giemsa and differential cell counts of pulmonary inflammatory cells (macrophages, neutrophils, lymphocytes, and eosinophils) were determined using standard morphological criteria and counting 3 × 100 cells per cytospin preparation. The total number of each cell type was then calculated and expressed as number of cells per mL of BAL fluid.

### Histological evaluation of the chronic allergen-challenged lungs

Following BAL, the lungs were inflated with 4% paraformaldehyde solution to a pressure of 20 cmH_2_O without removing the lungs from the chest. After 1 h the trachea was tied off, the lungs were stored at 4°C overnight in 4% paraformaldehyde, then washed several times in ethanol and stored in 70% ethanol at 4°C until time for embedding. After embedding in paraffin, the tissue was cut into 5 μm sections and mounted on positively charged slides. To assess inflammatory cell infiltration the sections were deparaffinized, dehydrated, and stained with hematoxylin and eosin (H&E). H&E stained sections were examined by bright field microscopy (Nikon Eclipse TS100, Nikon Instruments Inc., Melville, N.Y, USA) and images were captured with a Nikon DS digital camera system (Tekno Optik AB, Stockholm, Sweden).

### Statistical analysis

Results are presented as mean ± standard error of mean (SEM). Statistical significance was assessed by parametric methods using two-way analysis of variance (ANOVA) to analyze differences between groups, followed by Bonferroni post hoc test. When appropriate, one-way ANOVA or Student's unpaired t-test was used. A statistical result with *P *< 0.05 was considered significant. Statistical analysis and preparations of graphs were performed with GraphPad Prism (version 4.0 GraphPad software Inc., San Diego, CA, USA).

## Results

### Healthy mice

**Figure 2:**
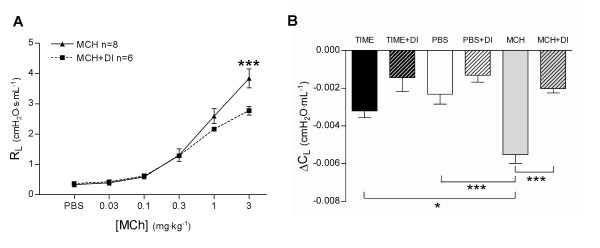
Effects of deep inspirations (DI) in healthy mice; (**A**) lung resistance (R_L_) in mice given incremental doses of methacholine (MCH group), and (**B**) the effect of DI on lung compliance (C_L_) presented as ΔC_L_. Values are mean ± SEM, * *P *< 0.05, ** *P *< 0.01, *** *P *< 0.001.

MCh increased R_L_, from baseline 0.33 ± 0.01 to 3.8 ± 0.3 cmH_2_O·s·mL^-1 ^(*P *< 0.001) at the highest dose of MCh (Figure 2A). DI significantly reduced the maximum R_L _from 3.8 ± 0.3 to 2.8 ± 0.1 cmH_2_O·s·mL^-1 ^(*P *< 0.001, Figure 2A). R_L _did not change from baseline in TIME or PBS groups, (no MCh provocation), with or without DI (*P *> 0.05).

C_L _was measured immediately before injections of PBS or MCh. In the TIME group, receiving no i.v. injections and no DI, C_L _decreased by 9.3 ± 0.8% from baseline to the last measurement point (ΔC_L_, Figure 2B). A similar decline was seen in the PBS group, receiving PBS injections without DI, where C_L _decreased by 6.9 ± 1.6% (*P *> 0.05, Figure 2B). In the MCH group, receiving incremental doses of MCh without DI, C_L _decreased by 15.9 ± 1.5%, the decline being significantly larger than in the TIME and PBS groups (*P *< 0.05 and *P *< 0.001 respectively, Figure 2B). DI significantly protected against the reduction in C_L _in the MCH+DI group, where the decline in C_L _was attenuated to 5.6 ± 0.6% (*P *< 0.0001, Figure 2B). Although displaying a tendency to protection, DI had no significant attenuating effect on the decrease in C_L _in either the TIME+DI (4.0 ± 1.9%, *P *> 0.05) or the PBS+DI group (3.8 ± 1.1%, *P *> 0.05, Figure 2B).

### Bronchoalverolar lavage and histology

Mice undergoing the 17-day or 98-day ovalbumin challenge protocol, the OVA'17 and OVA'98 group respectively, had clear signs of airway inflammation compared to control animals. OVA'17 group had approximately a 6-fold increase in total BAL cell count and OVA'98 had a 5-fold increase compared to control groups (both *P *< 0.001). Animals in the OVA'17 had a significant higher BAL cell count than OVA'98 (*P *< 0.03). Differential BAL cell count confirmed an inflammatory profile with markedly increased counts of macrophages, eosinophils, neutrophils, and lymphocytes in both acute and chronic challenged OVA groups. The OVA'17 animals had a higher number of eosinophils than OVA'98 animals (Table 1).

**Table 1:** Differential cell counts in bronchial alveolar lavage from animals having undergone an ovalbumin challenge protocol (OVA'17 and OVA'98) or a control protocol with phosphate buffered saline (PBS'17 and PBS'98).

	PBS'17 (n = 15)	OVA'17 (n = 17)	PBS'98 (n = 11)	OVA'98 (n = 11)
*Macrophages*	73 600 ± 4 100	147 000 ± 7 500 ^¤^	68 000 ± 5 500	168 000 ± 10 700 *
*Eosinophils*	0	222 100 ± 39 700 ^¤^	0	76 000 ± 26 500 *
*Neutrophils*	2 300 ± 500	3 900 ± 2 100	2 500 ± 2 000	52 500 ± 11 000 *
*Lymphocytes*	9100 ± 2400	23 000 ± 3 500 ^¤^	900 ± 350	23 500 ± 6 500 *

Values are mean ± SEM. ^¤^*P *< 0.05 vs. PBS'17, * *P *< 0.05 vs. PBS'98.

**Figure 3:**
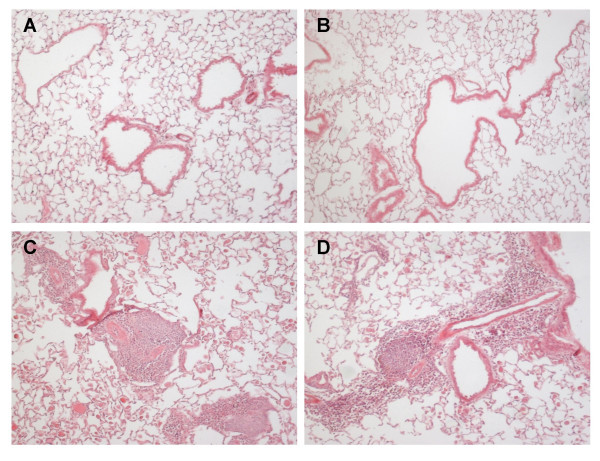
Representative histological sections (hematoxylin and eosin stained) from healthy control animals in the PBS'98 group (picture A and B) and from animals having undergone a 98-day ovalbumin challenge protocol, the OVA'98 group (picture C and D). Examination of sections from OVA'98 animals revealed a significant inflammation surrounding the airways and within the alveolar spaces. PBS'98 did not show any signs of inflammation.

OVA'98 group had also clear signs of remodeling, light microscopic examination of hematoxylin and eosin sections from OVA'98 and PBS'98 animals revealed an eosinophilic inflammation in the OVA-treated animals with a patchy distribution of eosinophils surrounding the airways and within the alveolar spaces. OVA'98 animals also revealed a significantly increased perivascular inflammation (Figure 3).

### Acute allergen-challenged mice

#### Lung resistance and compliance

**Figure 4:**
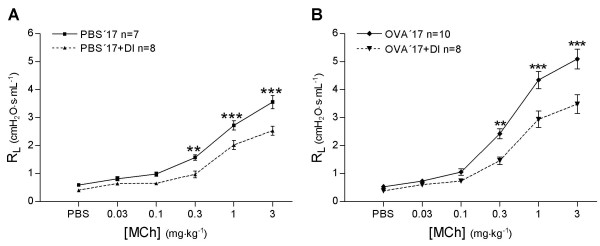
The effect of deep inspirations (DI) on lung resistance (R_L_) in healthy mice (PBS'17) and in animals with acute airway inflammation (OVA'17 group). Values are mean ± SEM, ** *P *< 0.01, *** *P *< 0.001.

In PBS'17 mice, MCh induced bronchoconstriction with a maximum R_L _of 3.6 ± 0.2 cmH_2_O·s·mL^-1^. After DI, R_L _was significantly lower, 2.5 ± 0.2 cmH_2_O·s·mL^-1 ^(*P *< 0.0001, Figure 4A). In OVA'17 mice, MCh induced bronchoconstriction with a maximum R_L _of 5.1 ± 0.3 cmH_2_O·s·mL^-1^. After DI, R_L _was significantly lower, 3.5 ± 0.3 cmH_2_O·s·mL^-1 ^(*P *< 0.0001, Figure 4B). In the OVA'17 group, MCh induced higher bronchoconstriction than the PBS'17 group, (*P *< 0.0001).

**Figure 5:**
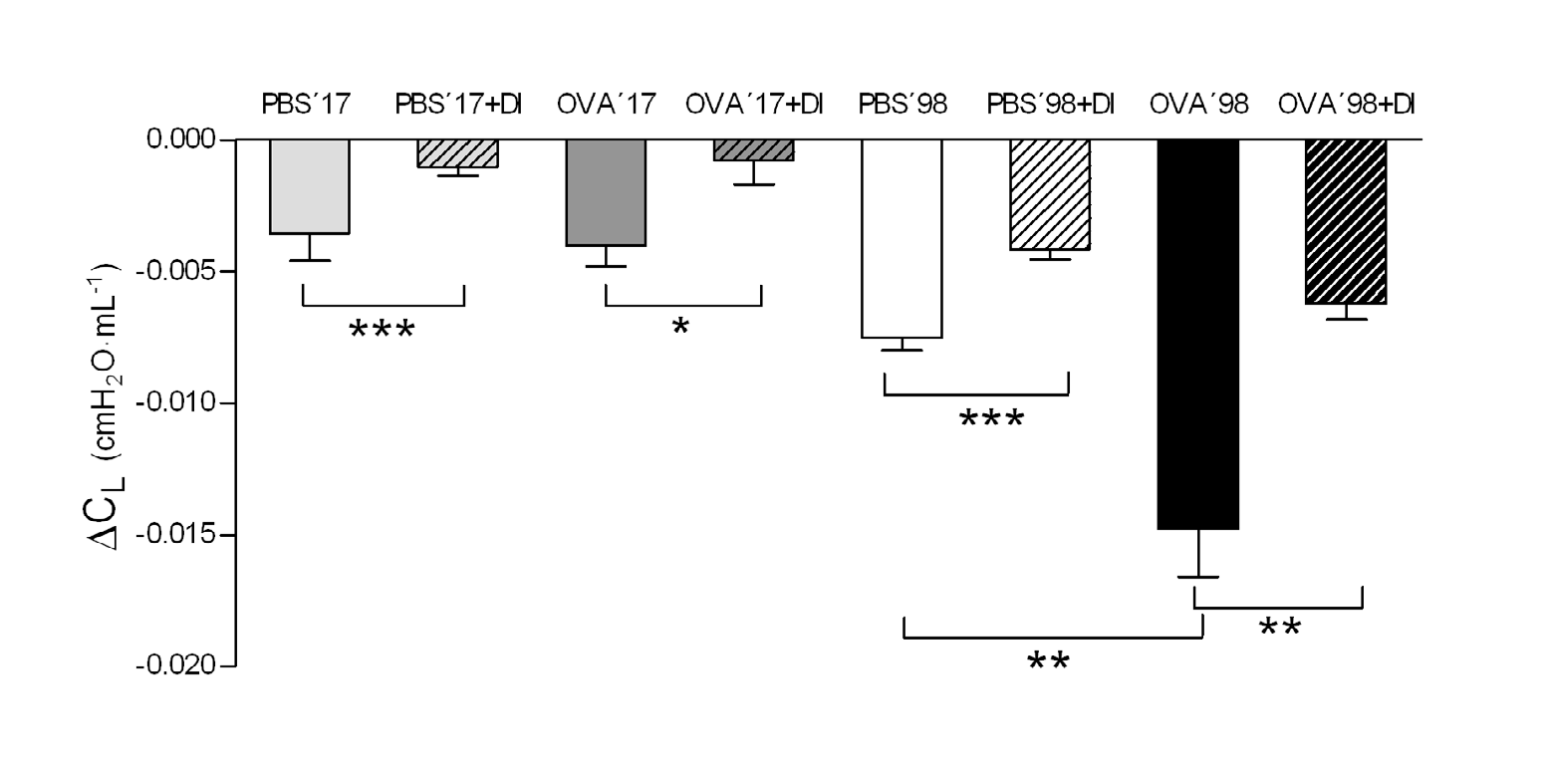
The effect of deep inspirations (DI) on lung compliance (C_L_) presented as ΔC_L_. DI attenuated the fall in ΔC_L _in both healthy mice (PBS'17) and in mice with acute airway inflammation (OVA'98). Mice with chronic airway inflammation, the OVA'98 group, had significantly larger fall in ΔC_L _than healthy control animals, the PBS'98 group. DI attenuated the fall in ΔC_L _in both groups, OVA'98 and PBS'98. Values are mean ± SEM, * *P *< 0.05, ** *P *< 0.01, *** *P *< 0.001.

In the PBS'17 group, C_L _decreased by 12.5 ± 3.2% from baseline to the last dose of MCh (Figure 5). Animals treated with DI, the PBS'17+DI group, had a significantly smaller decrease in C_L _(2.5 ± 1.6%, *P *< 0.05). In the OVA'17 group without DI, the decrease in C_L _was larger than in the PBS-treated animals (15.9 ± 2.3%, NS, Figure 5). In OVA-treated animals receiving DI, the OVA'17+DI group, the decrease in C_L _was largely prevented (2.7 ± 3.4%, *P *< 0.001, Figure 5).

#### Peripheral lung mechanics

During bronchial reactivity assessment the 4 s perturbation of forced oscillation (Prime 4) before each dose of PBS and MCh revealed significant differences in Newtonian resistance (R_N_) between OVA'17 and PBS'17 groups (23.3 ± 3.6% and 8.6 ± 4.5% respectively, *P *< 0.01). Treating animals with DI significantly lowered R_N _at each dose of PBS and MCh in OVA'17 group (OVA'17+DI, 10.5 ± 2.8%, *P *< 0.01). DI did not have any effect in the PBS'17 group (PBS'17+DI, 8.2 ± 3.9%, *P *> 0.05).

In the PBS'17 group, tissue elastance (H) increased by 9.4 ± 4.6% from baseline to the last dose of MCh. There was no protective effect on H in animals treated with DI, PBS'17+DI group. In the OVA'17 group without DI, H was two times higher than in the PBS'17 group (20.7 ± 3.1%, *P *< 0.0001). In the OVA'17+DI group, DI largely prevented the increase in H (8.5 ± 1.9%, *P *< 0.0001). There were no differences in tissue damping (G) in the PBS'17 group and the OVA'17 group (26.5 ± 4.4% and 14.5 ± 4.5% respectively, *P *> 0.05). DI prevented the increase in G in the OVA'17 group but not in the PBS'17 group (OVA'17+DI, 15.0 ± 2.2%, *P *< 0.05 and PBS'17+DI 4. 7 ± 3.1%, NS)

### Chronic allergen-challenged mice

#### Lung resistance and compliance

**Figure 6:**
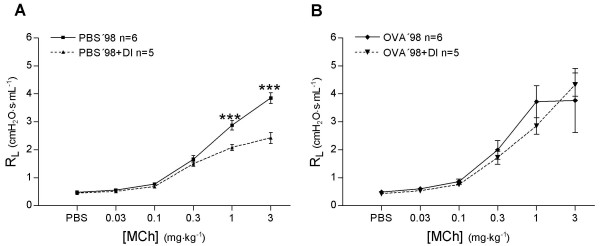
The effect of deep inspirations (DI) on lung resistance (R_L_) in healthy mice (PBS'98) and in mice with chronic airway inflammation (OVA'98 group). Values are mean ± SEM, *** *P *< 0.001.

In PBS'98 mice, MCh induced bronchoconstriction with a maximum R_L _of 3.8 ± 0.2 cmH_2_O·s·mL^-1^. After DI, R_L _was significantly lower, 2.4 ± 0.2 cmH_2_O·s·mL^-1 ^(*P *< 0.001, Figure 6A). This protective effect of DI against bronchoconstriction was totally abolished in OVA treated mice (OVA'98, 3.7 ± 1.1 cmH_2_O·s·mL^-1 ^and OVA'98+DI, 4.3 ± 0.4 cmH_2_O·s·mL^-1 ^respectively, *P *> 0.05, Figure 6B).

In the PBS'98 group, C_L _decreased by 18.1 ± 1.2% from baseline to the last dose of MCh (Figure 5). Animals treated with DI, the PBS'98+DI group, had a significantly smaller decrease in C_L _(9.7 ± 1.0%, *P *< 0.001). In the OVA'98 group without DI, the decrease in C_L _was more than double that in PBS-treated animals (44.1 ± 6.6%, *P *< 0.001, Figure 5). In OVA-treated animals receiving DI, the OVA'98+DI group, the decrease in C_L _was largely prevented (14.3 ± 1.3%, *P *< 0.001).

#### Peripheral lung mechanics

**Figure 7:**
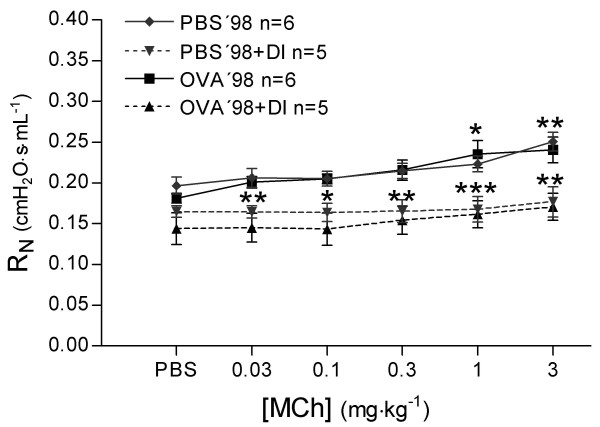
Measurements of Newtonian resistance (R_N_) were performed with forced oscillation technique (Prime 4 perturbation, Zrs measurements) before each injection of phosphate buffered saline or methacholine. *P *values for each significant R_N _value for each group; * *P *< 0.05, ** *P *< 0.01, *** *P *< 0.001 vs. same group without DI. Values are mean ± SEM.

**Figure 8:**
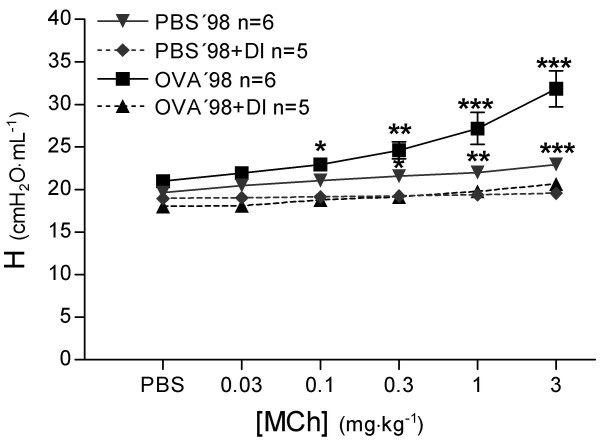
Measurements of tissue elastance (H) were performed with forced oscillation technique (Prime 4 perturbation, Zrs measurements) before each injection of phosphate buffered saline or methacholine. Values are mean ± SEM, * *P *< 0.05, ** *P *< 0.01, *** *P *< 0.001 vs. all other groups.

During bronchial reactivity assessment the 4 s perturbation of forced oscillation (Prime 4) before each dose of PBS and MCh revealed no significant differences in R_N _between OVA'98 and PBS'98 groups. Treating animals with DI significantly lowered R_N _at each dose of PBS and MCh in both groups (*P *< 0.0001, Figure 7). In the PBS'98 group, tissue elastance (H) increased by 16.7 ± 2.3% from baseline to the last dose of MCh (Figure 8). Animals treated with DI, PBS'98+DI group, had a significantly smaller increase in H (3.5 ± 2.0%, *P *< 0.0001). In the OVA'98 group without DI, H was three times higher than in the PBS'98 group (51.1 ± 7.5%, *P *< 0.0001). In the OVA'98+DI group, DI largely prevented the increase in H (14.7 ± 1.1%, *P *< 0.0001).

**Figure 9:**
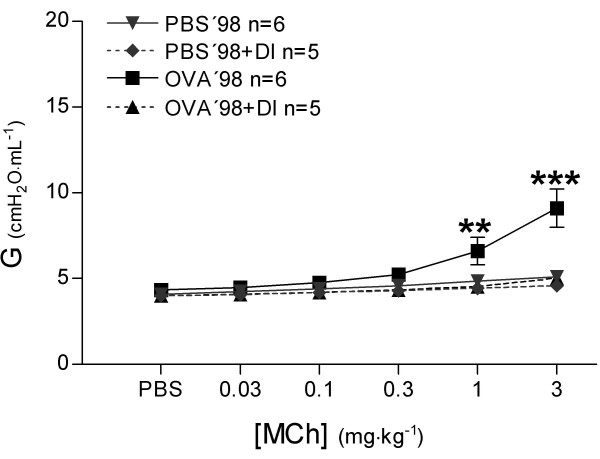
Measurements of tissue damping (G) were performed with forced oscillation technique (Prime 4 perturbation, Zrs measurements) before each injection of phosphate buffered saline or methacholine. Values are mean ± SEM, ** *P *< 0.01, *** *P *< 0.001 vs. all other groups.

In the OVA'98 group without DI, the increase in tissue damping (G) (Figure 9) from baseline was four times greater than in the PBS'98 group (108.1 ± 20% and 25.9 ± 4.97%, respectively, *P *< 0.0001). In the OVA'98+DI group, DI largely prevented the increase in tissue damping (25.0 ± 1.2%, *P *< 0.0001), while there were no differences in tissue damping between the PBS'98 and PBS'98+DI groups.

## Discussion

We have investigated the effects of deep inspirations (DI) in healthy mice, in mice with acute airway inflammation and in mice with chronic airway inflammation and remodeling. Our major findings are that: 1) DI had a marked effect on lung resistance after MCh-challenge in healthy mice and in acute allergen-challenged mice, but not in mice with chronic inflammation; 2) DI protects against the decrease in lung compliance that occurs both spontaneously over time and after intravenous injections of PBS or MCh; 3) DI has a major impact on peripheral airway and tissue physiology, protecting against MCh-induced increases in tissue elastance (H) in both animals with acute and chronic inflammation and also in healthy mice undergoing the 98-day protocol; 4) DI totally abolishes MCh-induced increases in tissue damping (G) seen in mice with acute and chronic inflammation.

This mouse model has potential value for defining mechanisms and sites of action of DI and our goals were to investigate if this mouse model could be used to identify the site of action of DI. We have implemented both the constant phase model (the low-frequency oscillation technique) and the single compartment model to characterize the effect of a DI. The constant phase model has the capacity to partition the respiratory properties into central and peripheral airways and also pure tissue properties [15,17,19]. In this study animals were of varying age depending on the duration of the different protocols. This could have possible effects on mouse lung mechanics [20-23], we solved this by having matched controls.

The airway protective effects of DI are similar to what has also been seen in other animal studies [24-27] and in humans [5-7,28]. The mechanisms underlying this bronchoprotective effect are not clear, but several hypotheses have been put forward as to how DI confers bronchoprotection [9], in which the main mechanisms have been suggested to be neural, nitric oxide (NO)-mediated, or mechanical. Scichilone et al. [7] suggested that DI could reduce bronchoconstriction through inhibition of cholinergic tone or activation of nonadrenergic, noncholinergic (NANC) system, and it has been suggested that airway stretch could cause release of substances such as NO [29] or cyclooxygenase products [30]. Mechanical explanations involve different theories, the simplest one being that stretching airway smooth muscle disrupts cross bridges, thereby reducing force generation. Fredberg et al. [31,32] suggested that asthmatic smooth muscle becomes "frozen" due to excessive latch bridge formation and that DI may detach these latch bridges, which provides an opportunity for normal cross-bridges. On the other hand, Gunst and co-workers [33,34] contend that cross-bridge properties cannot account for this, and that it is rather due to the plastic organization of contractile filaments in smooth muscle, allowing for adaptation to stretch [34]. This idea is in line with Wang and Paré [9] who proposed that DI initiate an adaptive process involving dissembly of contractile filaments, thereby allowing for reorganization of the contractile apparatus and better adaptation to the new smooth muscle cell length. In spite of recent investigations and new theories on the behavior of smooth muscle cells in response to stretch and mechanical forces [35-37], the cellular and subcellular mechanisms behind DI and bronchial responsiveness remain undefined. The current study provides a model for further investigation of the mechanisms.

Using short acute OVA challenge protocols [38], mice develop inflammation almost completely localized to the proximal airways, while chronic exposure to OVA leads to inflammation throughout the lung [39,40]. In the current study, mice were subjected to a 1-week or a 12-week OVA inflammation protocol and we found clear signs of inflammation and after the 12-week protocol there was also airway remodeling. Our results indicate that our 98-day long chronic inflammation model resembles human asthma more than an acute model does because of more peripheral inflammation in the lung after chronic challenge. When Wegmann [39] ran a similar protocol, chronic inflammation and remodeling were seen to involve peripheral airways, compared with acute inflammation that mainly involved proximal airways. Xisto et al [40] found inflammatory cell infiltration and remodeling of the central as well as the peripheral airways and lung parenchyma after a chronic inflammation protocol. Contrary to what Wegmann [39] and Xisto [40] reported, we could not detect any increased responsiveness to MCh in the chronically inflamed animals not receiving DI as compared with healthy mice and mice with acute airway inflammation. Possible explanations for this may be due to the use of a shorter OVA protocol [40] or to differences between assessing airway function with body-plethysmography [41] and our measurements of lung resistance. While cautiously interpreting responses based on the body plethysmography technique and refraining from directly comparing enhanced pause system and lung resistance [18,42], there is in a study by McMillan et al [1] a trend toward less reactivity after a long term chronic OVA-protocol that resembles our findings. Another explanation to our findings in the chronic inflammation could be that these animals induced a tolerance against OVA [43] and this could lead to a decreased responsiveness to MCh. Our results are also in line with human studies, where airway response to MCh is similar in healthy and asthmatic subjects when no DI is allowed [13], a phenomenon directly linked to narrowing of the conducting airways [44]. This has led us to believe that our mouse model of chronic airway inflammation closely resembles human asthma with respect to several points. Our present results show that DI protects from MCh-induced increase in lung resistance in healthy mice and in acute airway inflammation, but not in mice with chronic inflammation. The lack of protective effect against increased lung resistance in chronically inflamed mice is in line with human studies where DI gives asthmatic patients no protection against MCh-induced bronchoconstriction [5,6].

Most investigations of murine models of airway inflammation have focused on bronchial responsiveness and remodeling of more central airways. Recent reports show that the peripheral airways and parenchyma play a more important role in pathophysiology than expected. Lundblad et al [45] and Wagers et al [46] have recently shown that increased airway reactivity in OVA inflamed mice is entirely due to exaggerated closure of peripheral airways and that excessive narrowing is due to purely geometric reasons.

Inflammation of distal airways and lung parenchyma directly affects lung physiology by increasing tissue elastance and resistance, as well as by elevating pulmonary static and dynamic elastance [40]. In our current study, acute inflammation increased lung resistance and reduced lung compliance. There was no effect on tissue damping but an effect on tissue elastance. When applying DI before MCh-challenges, we saw a strong protective effect on lung resistance and lung compliance. DI had a significant protective effect on tissue elastance, while tissue damping was already low and was not altered by DI. However, chronic inflammation reduced lung compliance, while increasing tissue elastance and tissue damping. When applying DI before MCh-challenges, we saw stronger protective effects on these peripheral parameters in animals with chronic inflammation than in the acute inflammation. In healthy animals, DI had a significant protective effect on lung compliance and tissue elastance, while tissue damping was already low and was not altered by DI. This indicates that DI has a stronger effect on peripheral tissue in the chronic airway inflammation and that the protective effect of DI on lung resistance is greater in acute airway inflammation. Our results in the chronic airway inflammation are in line with those of Schweitzer et al [24], who showed that DI in Brown Norway rats, protected against MCh-induced increases in respiratory system elastance, but not resistance. Similar results were previously found by Hirai and Bates [25], who showed that DI, in healthy Sprague-Dawley rats, was neither bronchodilatory nor bronchoprotective, but indeed had a significant effect on both tissue damping and tissue elastance.

## Conclusion

In summary, we have found that presence of DI may blunt bronchoconstriction of central airways in healthy mice and in acute airway inflammation, but not when chronic inflammation is present. We have presented a murine OVA model that in many ways resembles human chronic airway inflammation. Many human studies suggest that DI is not bronchoprotective in asthmatic subjects, which is in line with our current findings in the chronic inflammation model. However, our present results point to very potent protective effects in the peripheral parts of the chronically inflamed murine lung and it is conceivable that this could also play a major role on overall lung health in asthma patients. This model of chronic airway inflammation should pave the way for investigations of mechanisms that may help identify new targets for therapies in chronic airway inflammation and asthma.

## Competing interests

The author(s) declare that they have no competing interests.

## Authors' contributions

SJ carried out the animal experiments and drafted the manuscript. LS carried out the histological evaluation and the cellular data. ML carried out animal experiments. JH, SED and GH participated in the study design, coordination and helped to draft the manuscript. All authors read and approved the final manuscript.

## References

[B1] McMillan SJ, Lloyd CM (2004). Prolonged allergen challenge in mice leads to persistent airway remodelling. Clin Exp Allergy.

[B2] Cui ZH, Skoogh BE, Pullerits T, Lotvall J (1999). Bronchial hyperresponsiveness and airway wall remodelling induced by exposure to allergen for 9 weeks. Allergy.

[B3] Brown RH, Mitzner W (2001). Airway response to deep inspiration: role of inflation pressure. J Appl Physiol.

[B4] Brusasco V, Crimi E, Barisione G, Spanevello A, Rodarte JR, Pellegrino R (1999). Airway responsiveness to methacholine: effects of deep inhalations and airway inflammation. J Appl Physiol.

[B5] Kapsali T, Permutt S, Laube B, Scichilone N, Togias A (2000). Potent bronchoprotective effect of deep inspiration and its absence in asthma. J Appl Physiol.

[B6] Scichilone N, Kapsali T, Permutt S, Togias A (2000). Deep inspiration-induced bronchoprotection is stronger than bronchodilation. Am J Respir Crit Care Med.

[B7] Scichilone N, Permutt S, Togias A (2001). The lack of the bronchoprotective and not the bronchodilatory ability of deep inspiration is associated with airway hyperresponsiveness. Am J Respir Crit Care Med.

[B8] Sundblad BM, Larsson K (2002). Effect of deep inhalations after a bronchial methacholine provocation in asthmatic and non-asthmatic subjects. Respir Med.

[B9] Wang L, Pare PD (2003). Deep inspiration and airway smooth muscle adaptation to length change. Respir Physiol Neurobiol.

[B10] Fredberg JJ (2001). Airway obstruction in asthma: does the response to a deep inspiration matter?. Respir Res.

[B11] Jensen A, Atileh H, Suki B, Ingenito EP, Lutchen KR (2001). Selected contribution: airway caliber in healthy and asthmatic subjects: effects of bronchial challenge and deep inspirations. J Appl Physiol.

[B12] Fish JE, Ankin MG, Kelly JF, Peterman VI (1981). Regulation of bronchomotor tone by lung inflation in asthmatic and nonasthmatic subjects. J Appl Physiol.

[B13] Skloot G, Permutt S, Togias A (1995). Airway hyperresponsiveness in asthma: a problem of limited smooth muscle relaxation with inspiration. J Clin Invest.

[B14] Irvin CG, Bates JH (2003). Measuring the lung function in the mouse: the challenge of size. Respir Res.

[B15] Hantos Z, Daroczy B, Suki B, Nagy S, Fredberg JJ (1992). Input impedance and peripheral inhomogeneity of dog lungs. J Appl Physiol.

[B16] Gomes RF, Shen X, Ramchandani R, Tepper RS, Bates JH (2000). Comparative respiratory system mechanics in rodents. J Appl Physiol.

[B17] Tomioka S, Bates JH, Irvin CG (2002). Airway and tissue mechanics in a murine model of asthma: alveolar capsule vs. forced oscillations. J Appl Physiol.

[B18] Bates JH, Irvin CG (2003). Measuring lung function in mice: the phenotyping uncertainty principle. J Appl Physiol.

[B19] Bates JH, Lutchen KR (2005). The interface between measurement and modeling of peripheral lung mechanics. Respir Physiol Neurobiol.

[B20] Bozanich EM, Janosi TZ, Collins RA, Thamrin C, Turner DJ, Hantos Z, Sly PD (2007). Methacholine responsiveness in mice from 2 to 8 wk of age. J Appl Physiol.

[B21] Bozanich EM, Collins RA, Thamrin C, Hantos Z, Sly PD, Turner DJ (2005). Developmental changes in airway and tissue mechanics in mice. J Appl Physiol.

[B22] Busse PJ, Zhang TF, Srivastava K, Schofield B, Li XM (2007). Effect of ageing on pulmonary inflammation, airway hyperresponsiveness and T and B cell responses in antigen-sensitized and -challenged mice. Clin Exp Allergy.

[B23] Hirai T, Hosokawa M, Kawakami K, Takubo Y, Sakai N, Oku Y, Chin K, Ohi M, Higuchi K, Kuno K (1995). Age-related changes in the static and dynamic mechanical properties of mouse lungs. Respir Physiol.

[B24] Schweitzer C, Demoulin B, Bello G, Bertin N, Leblanc AL, Marchal F (2006). Deep inhalation prevents the respiratory elastance response to methacholine in rats. Pediatr Res.

[B25] Hirai T, Bates JH (2001). Effects of deep inspiration on bronchoconstriction in the rat. Respir Physiol.

[B26] Chapman RW, Skeans S, Lamca J, House A, Hey JA, Celly C (2005). Effect of histamine, albuterol and deep inspiration on airway and lung tissue mechanics in cynomolgus monkeys. Pulm Pharmacol Ther.

[B27] Gunst SJ, Shen X, Ramchandani R, Tepper RS (2001). Bronchoprotective and bronchodilatory effects of deep inspiration in rabbits subjected to bronchial challenge. J Appl Physiol.

[B28] Skloot G, Togias A (2003). Bronchodilation and bronchoprotection by deep inspiration and their relationship to bronchial hyperresponsiveness. Clin Rev Allergy Immunol.

[B29] Bannenberg GL, Gustafsson LE (1997). Stretch-induced stimulation of lower airway nitric oxide formation in the guinea-pig: inhibition by gadolinium chloride. Pharmacol Toxicol.

[B30] Gao Y, Vanhoutte PM (1993). Responsiveness of the guinea pig trachea to stretch: role of the epithelium and cyclooxygenase products. J Appl Physiol.

[B31] Fredberg JJ, Inouye D, Miller B, Nathan M, Jafari S, Raboudi SH, Butler JP, Shore SA (1997). Airway smooth muscle, tidal stretches, and dynamically determined contractile states. Am J Respir Crit Care Med.

[B32] Fredberg JJ, Inouye DS, Mijailovich SM, Butler JP (1999). Perturbed equilibrium of myosin binding in airway smooth muscle and its implications in bronchospasm. Am J Respir Crit Care Med.

[B33] Gunst SJ (1983). Contractile force of canine airway smooth muscle during cyclical length changes. J Appl Physiol.

[B34] Shen X, Wu MF, Tepper RS, Gunst SJ (1997). Mechanisms for the mechanical response of airway smooth muscle to length oscillation. J Appl Physiol.

[B35] Gunst SJ, Fredberg JJ (2003). The first three minutes: smooth muscle contraction, cytoskeletal events, and soft glasses. J Appl Physiol.

[B36] Fabry B, Fredberg JJ (2003). Remodeling of the airway smooth muscle cell: are we built of glass?. Respir Physiol Neurobiol.

[B37] Deng L, Trepat X, Butler JP, Millet E, Morgan KG, Weitz DA, Fredberg JJ (2006). Fast and slow dynamics of the cytoskeleton. Nat Mater.

[B38] Hjoberg J, Shore S, Kobzik L, Okinaga S, Hallock A, Vallone J, Subramaniam V, De Sanctis GT, Elias JA, Drazen JM, Silverman ES (2004). Expression of nitric oxide synthase-2 in the lungs decreases airway resistance and responsiveness. J Appl Physiol.

[B39] Wegmann M, Fehrenbach H, Fehrenbach A, Held T, Schramm C, Garn H, Renz H (2005). Involvement of distal airways in a chronic model of experimental asthma. Clin Exp Allergy.

[B40] Xisto DG, Farias LL, Ferreira HC, Picanco MR, Amitrano D, Lapa ESJR, Negri EM, Mauad T, Carnielli D, Silva LF, Capelozzi VL, Faffe DS, Zin WA, Rocco PR (2005). Lung parenchyma remodeling in a murine model of chronic allergic inflammation. Am J Respir Crit Care Med.

[B41] Hamelmann E, Schwarze J, Takeda K, Oshiba A, Larsen GL, Irvin CG, Gelfand EW (1997). Noninvasive measurement of airway responsiveness in allergic mice using barometric plethysmography. Am J Respir Crit Care Med.

[B42] Adler A, Cieslewicz G, Irvin CG (2004). Unrestrained plethysmography is an unreliable measure of airway responsiveness in BALB/c and C57BL/6 mice. J Appl Physiol.

[B43] Van Hove CL, Maes T, Joos GF, Tournoy KG (2007). Prolonged inhaled allergen exposure can induce persistent tolerance. Am J Respir Cell Mol Biol.

[B44] Brown RH, Croisille P, Mudge B, Diemer FB, Permutt S, Togias A (2000). Airway narrowing in healthy humans inhaling methacholine without deep inspirations demonstrated by HRCT. Am J Respir Crit Care Med.

[B45] Lundblad LK, Thompson-Figueroa J, Allen GB, Rinaldi L, Norton RJ, Irvin CG, Bates JH (2007). Airways Hyperresponsiveness in Allergically Inflamed Mice: The Role of Airway Closure. Am J Respir Crit Care Med.

[B46] Wagers S, Lundblad LK, Ekman M, Irvin CG, Bates JH (2004). The allergic mouse model of asthma: normal smooth muscle in an abnormal lung?. J Appl Physiol.

